# INDANA - International Network for Diet And Nutrition in Allergy

**DOI:** 10.1186/2045-7022-1-S1-P41

**Published:** 2011-08-12

**Authors:** Carina Venter, Berber Vlieg-Boerstra, Isabel Skypala

**Affiliations:** 1 University of Portsmouth, Portsmouth, UK; 2 University Medical Centre, Amsterdam, Netherlands; 3 The Royal Brompton Hospital, London, UK

## Background

The International Network for Diet and Nutrition in Allergy (INDANA) was established in 2009 by a group of dietitians/food scientists specialising in food allergies and intolerances.

## Aim

The aim of this organisation is to bring together food allergy professionals to bridge the gap in science between food hypersensitivity, immunology, nutrition and food science to improve the nutritional management of those living with food allergies and intolerances.

## Steering group and champions

The steering group consist of 17 members with representation from the USA, United Kingdom, Europe, Australia, New Zealand and South Africa. INDANA has four prominent international champions, namely Prof. Susan Prescott (Australia), Prof Gideon Lack (UK), Prof Steve Taylor (USA) and Prof Hugh Sampson (USA), who are supporting INDANA activities in the wider community of international allergy practice,.

## Affiliations

The group is currently affiliated to the EAACI and is working towards collaborative multi-professional activities such as education, audit and research. Further affiliations with other well known associations in the allergy field are also explored. INDANA also aims to unify practices and develop evidence-based guidelines and protocols for the diagnosis and nutritional management of patients who suffer from food hypersensitivity.

## Activities

In 2010, members of INDANA presented at the American Academy of Allergy, Asthma and Immunology (AAAAI) meeting in New Orleans as well as the EAACI meeting in London. Two members of INDANA are presenting at the Food Allergy and Anaphylaxis Meeting (EAACI) in Venice 2011.

## Future developments

INDANA members will be presenting at the AAAAI meeting in 2011 addressing Nutritional Considerations in Food Allergy and the EAACI meeting in June 2011 in a session entitled Dietary management of food allergy: addressing cultural differences. INDANA hopes to increase their current membership numbers over the next few years and are actively developing conference and educational programs for 2012.

## Membership

Membership is open to any health care professional with a relevant first degree working in the field of food allergy (http://www.indana-allergynetwork.org).

